# Whole Genome Sequencing Analysis of Effects of CRISPR/Cas9 in *Komagataella phaffii*: A Budding Yeast in Distress

**DOI:** 10.3390/jof8100992

**Published:** 2022-09-21

**Authors:** Veronika Schusterbauer, Jasmin E. Fischer, Sarah Gangl, Lisa Schenzle, Claudia Rinnofner, Martina Geier, Christian Sailer, Anton Glieder, Gerhard G. Thallinger

**Affiliations:** 1 bisy GmbH, Wuenschendorf 292, 8200 Hofstaetten, Austria; 2 Institute of Biomedical Imaging, Graz University of Technology, Stremayrgasse 16, 8010 Graz, Austria; 3 Institute of Biomedical Informatics, Graz University of Technology, Stremayrgasse 16, 8010 Graz, Austria; 4 OMICS Center Graz, BioTechMed Graz, Stiftingtalstraße 24, 8010 Graz, Austria

**Keywords:** CRISPR/Cas technology, non-conventional yeasts, genome analysis, non-homologous end joining

## Abstract

The industrially important non-conventional yeast *Komagataella phaffii* suffers from low rates of homologous recombination, making site specific genetic engineering tedious. Therefore, genome editing using CRISPR/Cas represents a simple and efficient alternative. To characterize on- and off-target mutations caused by CRISPR/Cas9 followed by non-homologous end joining repair, we chose a diverse set of CRISPR/Cas targets and conducted whole genome sequencing on 146 CRISPR/Cas9 engineered single colonies. We compared the outcomes of single target CRISPR transformations to double target experiments. Furthermore, we examined the extent of possible large deletions by targeting a large genomic region, which is likely to be non-essential. The analysis of on-target mutations showed an unexpectedly high number of large deletions and chromosomal rearrangements at the CRISPR target loci. We also observed an increase of on-target structural variants in double target experiments as compared to single target experiments. Targeting of two loci within a putatively non-essential region led to a truncation of chromosome 3 at the target locus in multiple cases, causing the deletion of 20 genes and several ribosomal DNA repeats. The identified *de novo* off-target mutations were rare and randomly distributed, with no apparent connection to unspecific CRISPR/Cas9 off-target binding sites.

## 1. Introduction

Genetic engineering of microbes has led towards promising developments in the production of pharmaceutical products, alternative fuels, and meat alternatives, among other important proteins and bulk chemicals [1,2,3,4]. The practical implementation of the optimal production strain, however, is regularly hampered by the demanding process of genetic engineering [5]. Many of the toolboxes recently developed around CRISPR/Cas (clustered regularly interspaced short palindromic repeats/CRISPR-associated protein) proved to be more efficient than conventional genetic tools [6,7,8,9,10]. This fantastic tool was derived from a natural defense mechanism in bacteria and archaea [11]. It facilitates the introduction of targeted double-strand DNA breaks (DSBs), which can trigger multiple DNA repair mechanisms in the cell. These include homologous recombination (HR), non-homologous end joining (NHEJ), and microhomology-mediated end joining (MMEJ) [10,12,13,14,15]. The main advantage of CRISPR/Cas over similar tools like TALENS [16] or zinc finger nucleases [17] is the simplicity of reprogramming it to a specific target [18,19]. Adapting a plasmid to a novel target can be as simple as exchanging 17–20 bp within the guide RNA (gRNA) [20].

Since their emergence, CRISPR-based methods have constantly been connected to concerns about off-targeting and other adverse effects. These have been extensively investigated in eukaryote model organisms such as mice and monkeys, or human cancer cell lines [21,22,23,24]. Though, some of the studies are limited to the analysis of variants arising at the targeted loci [12,18,24,25] and others are focusing on the effects on the whole genome [21,26,27,28,29,30,31,32,33]. One of the initial problems in applying CRISPR/Cas was off-target effects caused by unspecific binding of the gRNA [34], but more recent studies conclude that CRISPR/Cas, if well designed, does not lead to increased mutability [21,28]. Unwanted on-target effects have, by contrast, proven to be of greater concern. They were shown to be largely non-random, with the percentage of large deletions and other structural variants (SVs) accounting for up to 20% of repair outcomes [18,23,24,25]. The plethora of produced data has facilitated the development of multiple tools for gRNA design, which aim to minimize off-target effects and maximize on-target efficiency of different CRISPR-based methods. Some of the tools even predict the probability of frameshifts and SVs at the target site [35].

*Komagataella phaffii* (formerly *Pichia pastoris*) is a non-conventional yeast, which has recently become a very popular choice as production host for a diverse range of recombinant proteins and bulk chemicals. It is valued for its growth to extraordinarily high cell densities and high yield in the production of recombinant proteins [36,37], but it suffers from low rates of HR, therefore rendering targeted integrations or gene knockouts via HR very laborious [38,39,40]. Furthermore, due to the low success rate, these standard methods require the use of selectable markers. These can be removed in an additional step using site-specific recombinases (e.g., Cre-loxp, FLP-frt) [8,41], but these methods may still leave unwanted scars in the genome. Nonetheless, an extensive toolkit of *K. phaffii* platform strains and expression cassettes using diverse promoters, constitutive or induced, has been developed in recent years [39,42,43,44,45,46,47]. Introducing frameshift mutations via CRISPR is a convenient alternative to classical knockout strategies and the CRISPR/Cas tools designed for *K. phaffii* have shown striking targeting efficiencies reaching 70–100%, depending on the target [6,48,49,50]. However, some of the results raised suspicion about large on-target deletions or translocations in CRISPR/Cas-transformed *K. phaffii* clones [6].

Previous studies on CRISPR/Cas in *K. phaffii* have been restricted either to the analysis of phenotypical effects or to the sequencing of the CRISPR/Cas target loci based on PCR products. Hence, genome-wide effects of this genome editing tool are still unknown, as is the range of adverse on-target effects. We analyzed a range of CRISPR/Cas9 mediated mutations, generated by DSBs followed by error prone NHEJ repair in *K. phaffii,* on a genomic scale. We focused on generating a sound overview of events by choosing targets distributed over the whole genome and sequencing a diverse set of transformed colonies. Additionally, we explored the applicability of CRISPR/Cas9 to remove large stretches of the *K. phaffii* genome by targeting a putatively non-essential (NE) region. Using short-read, whole genome sequencing (WGS), we identified on-target and off-target effects in single and double target experiments.

## 2. Materials and Methods

### 2.1. Identification of Target Genes/Regions & Guide RNA Design

Genes with the potential to increase secretion of recombinant proteins in *K. phaffii* were identified by determining relevant gene ontology annotations in *S. cerevisiae* and consecutively finding genes with high similarity in *K. phaffii* (~70% identity based on a BLASTP search). The biological processes considered most interesting were “cell wall mannoprotein biosynthetic process” (GO:0000032), “regulation of fungal-type cell wall organization”, (GO:0060237) and “protein secretion” (GO:0009306). In total, 20 genes of interest were identified, of which 5 were chosen for a more detailed analysis of CRISPR/Cas9 on- and off-target effects, based on preliminary results and position in the genome (Table 1). Cereghino et al. have identified targets whose disruption might have positive effects on the secretion of the reporter protein beta galactosidase [51,52]. We have chosen to include 4 of those genes as targets and named them *BGS5*, *BGS7*, *BGS12* and *BGS13* in accordance with Cereghino et al. (Table 1). Due to the limited number of sequenced strains for these targets, we included them for analysis of off-target effects only.

To define a target region for a large knockout, essential genes of *K. phaffii* were identified by combining the genome annotation provided by Valli et al. [60] and the identified *S. cerevisiae* homologues in *K. phaffii,* together with the vast knowledge on gene viability in the Saccharomyces Genome Database (SGD) [61] using R/Bioconductor statistic software packages. We found 10 regions larger than 50 kb, which presumably contain no essential genes. We further inspected the two longest stretches identified for known essential genes in *K. phaffii.* We also looked for genes, which are reported as non-essential in *S. cerevisiae* due to duplication but might be essential in *K. phaffii*.

Three gRNAs per gene/region were designed using the CRISPR gRNA design tool from ATUM (Newark, CA, USA). To enhance the probability of frameshift mutations, which disrupt the respective protein function, only targets within the first 30–40% of each coding sequence were considered. The gRNAs, including all possible PAM motives (NGG), were blasted against known assemblies of *K. phaffii* (NCBI: txid460519) and only gRNAs which did not show any hits were considered for further use (Supplementary Table S2).

### 2.2. Strains and Constructs

Plasmids and strains used in this study, including their bisy strain collection numbers, are listed in Supplementary Table S1. Primers and other synthetic sequences for plasmid construction are listed in Supplementary Tables S2 and S3. Plasmid maps are included in the Supplementary Methods.

#### 2.2.1. Platform Strains

The platform strains *K. phaffii* BSYBG10_aox1_3S1K-CalB and *K. phaffii* BSYBG10_chr3ne_HygR are based on the commercially available strain *K. phaffii* BSYBG10 (bisy GmbH, Hofstaetten/Raab, AUT), which is a single colony streak-out of *K. phaffii* BG10 (BioGrammatics Inc., Carlsbad, CA, USA) [62]. Strain *K. phaffii UPP-C* (*K. phaffii* BSYBG11_pPpT4_PUPP_alpha_CalB) is based on strain *K. phaffii* BSYBG11, a commercially available *AOX1* knockout strain of BSYBG10.

Strain *K. phaffii* BSYBG10_3S1K-CalB was constructed by targeted integration of plasmid pBSY3S1K_intAOX1_CalB into the *AOX1* locus. pBSY3S1K_intAOX1_CalB is based on the commercial plasmid pBSY3S1K (bisy GmbH) and expresses a synthetic codon optimized gene, coding for the lipase B of *Candida antarctica* (CalB), as used by Vogl et al. [42], under the control of the P_CAT1_ promoter [42]. The plasmid backbone, as well as the P_CAT1_ promoter and the deletion variant of the *S. cerevisiae* mating factor alpha pre-pro-peptide for CalB secretion, were amplified from pBSY3S1K. The 5′ and 3′ *AOX1* homologous regions, with a length of 779 bp and 570 bp, respectively, were amplified from the genomic DNA of strain BSYBG10. Subsequently, Gibson Cloning was performed with all 5 fragments at once [63]. For integration into strain BSYBG10, the plasmid was linearized with SmiI. Selection of positive transformants occurred on YPD agar plates supplemented with 300 mg/L geneticin. Replication on agar plates during cultivation was done on BMM plates to ensure a mut^S^ phenotype, which confirmed the correct integration into the *AOX1* locus.

For strain *K. phaffii* BSYBG10_chr3ne_HygR, a cassette carrying a Hygromycin resistance under the control of the P_ILV5_ promoter [39] was integrated at the beginning of the identified stretch of non-essential genes of chromosome 3 (replacing pos. 2,169,490–2,169,713). The Hygromycin expression cassette, flanked by homologous stretches of ~1000 bp for targeted integration into the identified non-essential gene region, was generated by overlap extension PCR. The 5′ and 3′ homologous regions were amplified by PCR from strain BSYBG10. The Hygromycin marker including promoter and terminator were amplified from the commercially available plasmid pBSYBiEH (bisy GmbH). Specific primers, including short overlapping sequences to the corresponding fragments were used for amplification. Generated fragments were aligned and amplified by stepwise overlap extension PCR. After transformation of BSYBG10 using the generated resistance cassette, transformants were identified by growth on selective YPD agar plates containing 300 µg/mL Hygromycin. Targeted integration was verified by colony PCR.

Strain *K. phaffii* UPP-C was created by the random integration of plasmid pPpT4_PUPP_alpha_CalB into the genome of *K. phaffii* BSYBG11. This expression vector is based on the *K. phaffii*—*E. coli* shuttle vector pPpT4_S (NCBI JQ519690.1) and harbors a codon optimized DNA sequence coding for the *C. antarctica* lipase, CalB, under the control of the P_UPP_ promoter, a Zeocin resistance cassette for selection of positive transformants and the *S. cerevisiae* mating factor alpha pre-pro-peptide fused to the mature lipase for CalB secretion [44].

All three platform strains were whole genome sequenced as single colonies (as described under Genome sequencing and Analysis), to verify integration loci and single copy integrations of the used expression cassettes.

#### 2.2.2. CRISPR/Cas9 Plasmids

Single target and double target CRISPR/Cas9 plasmids were cloned as described by Weninger et al., using shuttle vector pPpT4_pHTX1-PARS1-hsCas9 (NCBI MW604246.1) [6,48]. All gRNA coding DNA sequences were ordered as synthetic DNA from TWIST (TWIST Bioscience, South San Francisco, CA, USA). For double target CRISPR/Cas9 plasmids, pPpT4_pHTX1-PARS1-hsCas9 was extended by a synthetic DNA element, which allows the expression of a second gRNA under the P_HHT2_ promoter [43,64]. The DNA element, which includes a P_HHT2_ promoter and a DAS2 terminator separated by a SmiI restriction site, was ordered from TWIST. For CRISPR/Cas9 plasmids used with BSYBG10_chr3ne_HygR, the expression of the Zeocin resistance was put under the control of promoter P_TEF1_ from *Ashbya gossypii* [65,66] in order to reduce sequence homology to the Hygromycin resistance expression cassette, which carries a P_ILV5_ promoter. The marker-cassette was inserted into the vector by Gibson isothermal assembly. After sequence verification by Microsynth AG, gRNAs were cloned gradually into the vector by Gibson isothermal assembly.

### 2.3. Transformation and Screening

Transformation of electrocompetent *K. phaffii* cells was performed following the condensed protocol of Lin-Cereghino et al. [67], using about 1 µg linearized DNA or 200 ng of plasmid DNA. After regeneration for 2–3 h in 1 mL YPD/Sorbitol (1:1), cells were plated on YPD (1% *w/v* yeast extract, 2% *w/v* peptone and 2% *w/v* glucose) plates (1.5% agar) containing 100 µg/mL Zeocin.

Transformations with plasmids for CRISPR/Cas9 based genome engineering were performed according to the method by Weninger et al. [6,48]. In short, after selection of positive transformants, cells are cultured on 96 well deep well plates (DWP) for 2 days in 250 µL YPD containing 100 µg/mL of Zeocin. For BSYBG10_chr3ne_HygR based colonies, the time given for CRISPR/Cas9 transformation was increased to 4 days. Plasmid curation was facilitated by transferring 5 µL of each well into fresh YPD containing DWPs and incubation for 48 h. Plasmid-loss was analyzed based on growth inability on selective YPD agar plates containing 100 µg/mL Zeocin. Strains based on BSYBG10_chr3ne_HygR were additionally stamped onto YPD agar plates containing 300 µg/mL Hygromycin to check for the loss of the Hygromycin resistance.

The screening of transformants by measuring CalB activity was adapted from the protocol described by Zhang and colleagues and further used elsewhere [68,69,70]. In brief, 250 µL BMG 1% were inoculated with a fresh single colony and incubated for at least 24 h (28 °C, 320 rpm). Feeding occurred by adding BMG media every 8 to 16 h to an absolute glycerol concentration of 0.25%. For harvesting, the cultures were centrifuged at 3220× *g* for 15 min (4 °C). Additionally, 20 µL of culture supernatant were mixed with 180 µL of the reaction solution. Absorption at 405 nm was measured for 3 min in 20 s intervals on a SpectraMax^®^ ABS Plus (Molecular Devices, San Jose, CA, USA), at room temperature, to follow esterase activity.

### 2.4. Genome Sequencing and Analysis

Initial sequencing of CRISPR/Cas9 transformants was performed on cell pellets derived from overnight cultures (ONCs) of single colonies. For a broad and systematic analysis of on- and off-target events, ten colonies per transformation, which were evenly spread across the CalB expression landscape, were sequenced in pooled cultures. For ONCs, single colonies were grown in 5 mL YPD overnight. For pooled sequencing, equal cell quantities were taken from 5 ONCs from the same experiment and mixed in one tube before centrifugation (about 250 µL at an OD_600_ of 6.0). The extraction of genomic DNA from cell pellets and all steps for Illumina sequencing were performed by Macrogen (Macrogen Inc., Seoul, South Korea). DNA was extracted using Maxwell^®^ Prokaryote/Eukaryote SEV DNA Purification Kit (Promega GmbH, Madison, WI, USA), followed by library preparation with either TruSeq DNA PCR-free or TruSeq DNA Nano (Illumina Inc., San Diego, CA, USA) kits with a target fragment size of 550 bp. Fragments were sequenced from both sides with 150 bp read length on either a NovaSeq 6000 or a HiSeq X (Illumina Inc., San Diego, CA USA; Supplementary Table S4). The targeted average read coverage for single colonies was 100-fold, and 300-fold for mixed cultures of five colonies. Quality checks of total genomic DNA and the prepared library were performed according to Macrogen’s standard procedures. Chosen targets were validated with colony PCRs and Sanger sequencing performed by Microsynth (Microsynth AG, Balgach, Switzerland). This information was used to correct on-target mutation results and clone number for human error during picking of clones and generating mixed cultures (Supplementary Tables S5–S7).

Reads were mapped to the reference using the Burrows–Wheeler Aligner (BWA-MEM) v0.7.17 [71]. As the reference sequence, we used the assembly of *K. phaffii* CBS7435 by Sturmberger et al. [62], including the 4 chromosomes and 2 killer plasmids, merged with the mitochondrial sequence published by Küberl et al. (NCBI LT962476.1-LT962479.1, MG491503.1 & MG491504.1, FR839632.1) [60,72]. Alignments were sorted, filtered for unmapped reads and duplicates were marked with Picard v2.20.3 (Available online: https://broadinstitute.github.io/picard (accessed on 21 July 2019)) and samtools v1.11 [73]. Single nucleotide variants (SNVs) and small insertions and deletions (InDels) were called based on GATK best practices [74]. Regions around small variants were realigned using GATK and consecutively called with the GATK Haplotype caller v4.2.0.0 [75]. For samples sequenced in pooled cultures, the assumed ploidy was set to 5, as we pooled 5 colonies for sequencing. The called InDels and SNVs were annotated for their effect on gene expression using SnpEff v4.3 [76]. Structural variants were called with GRIDSS v2.5.0 [77]. All types of variants were further annotated and filtered with R/Bioconductor statistic software packages as described in the Supplementary Methods. On-target mutations were additionally visually confirmed using the Integrative Genomics Viewer (IGV) [78]. To determine the relative read support for each on-target mutation, the mutant genotypes were reconstructed in SnapGene (Insightful Science, San Diego, CA, USA). Illumina reads were mapped onto the constructed multi-FASTA files containing all different genotypes with BWA-MEM. Alignments were filtered for unmapped and multimapped reads and sorted using samtools. Read support was counted at the exact position of the variant.

The genomic DNA for the *de novo* assembly of strain BSYBG10_LKO_B10 was prepared following the protocol for the preparation of yeast samples of the QIAGEN^®^ Genomic DNA Handbook (v June 2015, QIAGEN, Hilden, Germany). The quantities of the chemicals used were optimized for a mini-prep using the QIAGEN^®^ Genomic-tip 20/G, for DNA purification. DNA was quantified using the Qubit dsDNA BR assay (ThermoFisher, Vienna, Austria). Fragment sizes of isolated DNA were determined by pulsed field gel electrophoresis using a Pippin Pulse System (SAGE Bioscience, Biozym Biotech Trading GmbH, Vienna, Austria). The library was prepared using the SQK-LSK110 Ligation Sequencing Kit (Oxford Nanopore Technologies, Oxford, UK) and sequenced on a MinION device (ONT) using a single Flongle Flow Cell (FLO-FLG001, ONT). Bases were called using Guppy v5.0.17 (ONT) and the “fast-basecall” algorithm. The resulting reads, passing the Guppy QC, were assembled using Canu v2.2 [79] and polished by mapping Illumina reads to the draft assembly using BWA-MEM and consecutively running Pilon v1.24 [80]. The resulting assembly was compared to the reference assembly using NucDiff v2.0.3 [81].

### 2.5. Detection of CRISPR/Cas9 Off-Targets

We identified possible off-target binding sites of the gRNAs with CHOPCHOP [82], using the available reference for *K. phaffii* CBS7435. Additionally, we determined all possible off-target binding sites with up to ten mismatches with the R/Bioconductor statistical framework [83,84]. For off-target binding sites with less than 5 mismatches, we visually checked the mapped reads within IGV for possible mutations. Furthermore, we searched for occurrences of possible unspecific off-target binding sites of the used gRNAs within 30 bases up and downstream of the variant range. As a search pattern, we used the experiment specific gRNAs including either NGG or NAG as the PAM motive. We included all binding sites with up to 10 mismatches outside of the PAM motive. Mismatches within the PAM motive were not allowed. We used the R package Biostrings v2.60.2, and its functions *matchPattern* and *mismatch* [85], to find off-targets and define mismatch positions. Finally, only variants overlapping the defined off-target site, including the PAM motive were counted.

### 2.6. Transcriptome Sequencing and Analysis

Two clones, both harboring a chromosomal rearrangement, one from the simultaneous targeting of *MSG5-NCE102* and one from the targeting of *MSG5-FKS1* were chosen for transcriptome analysis. Colonies were grown in ONCs and immediately frozen at −80 °C after centrifugation. RNA samples were processed by Genewiz (Genewiz Azenta, Leipzig, Germany). RNA was extracted from cell pellets using Qiagen RNeasy Plus Mini Kit (Qiagen, Hilden, Germany) and library prep was performed with NEBNext^®^ Ultra™ II Directional RNA Library Prep Kit for Illumina (New England Biolabs Inc., Ipswich, MA, USA). Fragments were sequenced with a read length of 150 bp from both ends. Reads were mapped to the reference using the STAR aligner v2.7.5a [86].

### 2.7. Growth Analysis

Strains BSYBG10, BSYBG10_chr3ne_HygR, and BSYBG10_LKO_B10 were chosen for a comparative growth analysis. For precultures, 5 mL YPD were inoculated with a single colony and incubated at 110 rpm and 28 °C. Each strain was grown in triplicate, using 250 mL shake flasks with 50 mL buffered minimal glycerol 1% (BMG1), which were inoculated from the precultures to an OD_600_ of 0.1. Starting six hours after inoculation, OD_600_ was measured every two hours in technical triplicate. OD was measured from 200 µL of different dilutions (up to 1:20) with the SpectraMax^®^ ABS Plus (Molecular Devices, San Jose, CA, USA). Values were then blank corrected and multiplied by the dilution factor. Growth curve fitting was done in R using the package GrowthCurver [87]. We fitted one curve per biological sample and compared the resulting maximum growth rates using a one-tailed Wilcoxon signed rank test. A *p*-value equal or below 0.05 was considered statistically significant.

## 3. Results

In total, we analyzed the genomes of 146 CRISPR/Cas9 transformants of three different *K. phaffii* platform strains using paired-end Illumina sequencing. All unprocessed sequence data is available via ENA project PRJEB54592 (Available online: http://www.ebi.ac.uk/ena/data/view/PRJEB54592 (accessed on 19 August 2022)). For control experiments, *K. phaffii* strains resulting from transformations using CRISPR/Cas9 plasmids with no gRNA, as well as standard CRISPR/Cas9 plasmids with one gRNA, and plasmids for co-expression of two gRNAs were included. The gene targets were distributed across the whole genome to get an overview of CRISPR/Cas9 related on- and off-target effects.

**Figure 1:**
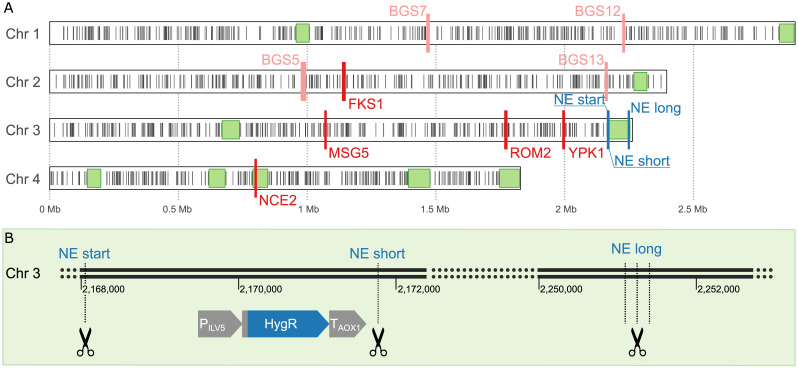
(**A**) Overview of all CRISPR targets and identified non-essential regions. Putatively non-essential (NE) regions with longer more than 50 kbp are shown as light green rectangles, while essential genes are depicted as black bars. The CRISPR targets for strain BSYBG10_chr3ne_HygR are shown in dark blue bars. Potential secretion-relevant targets for strain BSYBG10_3S1K_CalB based on homology to described effects in *S. cerevisiae* are shown in red and targets for strain UPP-C are shown in light red. (**B**) Detailed view of the 3′ end of chromosome 3 of strain *K. phaffii* BSYBG10_chr3ne_HygR, showing the location of the Hygromycin resistance expression cassette at the 5′ end of the NE region and the CRISPR targets aimed to remove the Hygromycin resistance cassette only (NE start–NE short) or the whole NE region (NE start–NE long).

We targeted nine genes which were suspected to be connected to protein secretion (Table 1), using *Candida antarctica* lipase B (CalB) as a reporter protein for phenotypic alterations. Four of these targets (*BGS5*, *BGS7*, *BGS12* and *BGS13*) were selected from a list determined in a study about β-galactosidase super-secretors (BGS) [52]. We identified further genes with a potential relation to protein secretion by searching for highly conserved *S. cerevisiae* orthologues in *K. phaffii*, for which *S. cerevisiae* genes are annotated to be part of one of the following biological processes: “Cell wall mannoprotein biosynthetic process”, “Regulation of fungal cell wall organization”, or “Protein secretion”. Finally, out of the *S. cerevisiae* homologues, we chose the genes *FKS1*, *MSG5*, *ROM2*, *YPK1* and *NCE102* for studying potential differences in single gRNA CRISPR/Cas9 transformations and double target experiments, since they are located on chromosomes 2, 3, and 4, respectively (Figure 1A, Table 1). For the double target experiments, we chose the target combinations *MSG5-FKS1*, *MSG5-YPK1*, and *MSG5-NCE102* using the same gRNAs as in single target experiments.

In addition, we explored the full extent of effects experienced in response to more drastic CRISPR/Cas9-mediated DSBs and deletions. By targeting a putatively non-essential region in the genome, we studied the consequences of targeted large deletions, without the use of a repair template for HR. We defined regions of at least 50 kbp in size, which most likely do not contain essential genes (Figure 1A). The largest such region is located at the 3′ end of chromosome 3 and comprises 20 genes (Supplementary Table S8). We shortened the region at the 5′ end to exclude the gene *PET8*, which encodes for an S-adenosylmethionine transporter of the mitochondrial inner membrane and was reported to be essential for respiratory growth [88]. The final region (LT962478.1:2,167,874-2,253,884) spanned about 85 kbp. To be able to quickly validate the success of the approach, we generated the platform strain BSYBG10_chr3ne_HygR by site-specific integration of a Hygromycin resistance cassette into *K. phaffii* strain BSYBG10, at the 5′ end of the non-essential region. The target loci for the CRISPR/Cas9 complex were located immediately before and after the Hygromycin resistance cassette (named NE start and NE short, respectively) in order to produce a clean knockout without using a repair template. Another target locus is located at the 3′ end of the selected non-essential region (named NE long), which should potentially trigger a deletion of the whole NE region (Figure 1B).

Initially, up to three single colonies per transformation experiment were sequenced individually to gain a first impression and obtain unambiguous results for the analysis of on- and off-target variants. To get a broader overview of the whole range of on- and off-target effects of CRISPR/Cas9 transformations on the genome of *K. phaffii*, we chose ten single colonies from each of the single target and double target experiments. The single target experiments targeted *FKS1*, *MSG5*, *ROM2*, *YPK1*, and *NCE102*; the double target experiments targeted the combinations *MSG5-FKS1*, *MSG5-YPK1*, and *MSG5-NCE102*. As a control, we sequenced ten single colonies from transformations with the empty CRISPR/Cas9 plasmid carrying no gRNA. For each experiment, two Illumina libraries were prepared from a cell pellet containing a pooled culture of five single colonies. To minimize any selection bias, we chose colonies for sequencing which showed a diverse range of CalB activity. Similarly, for the double target experiments aiming at the region containing NE-genes, we chose 20 single colonies for sequencing. From the first experiment, where we only targeted the introduced Hygromycin resistance cassette (targets NE start & NE short), we selected ten colonies which showed the ability to grow on plates containing Hygromycin after transformation with CRISPR/Cas9, and ten without that ability. From the double target experiments in which we targeted the complete NE region (NE start–NE long), we chose 15 single colonies which all had the ability to grow on plates containing Hygromycin. These 35 colonies were again pooled in groups of 5 for Illumina library construction. Two clones, which did not grow on Hygromycin containing plates and which showed a short deletion between NE start and NE short based on colony PCR of the locus, were sequenced individually.

### 3.1. On-Target Behavior

**Figure 2:**
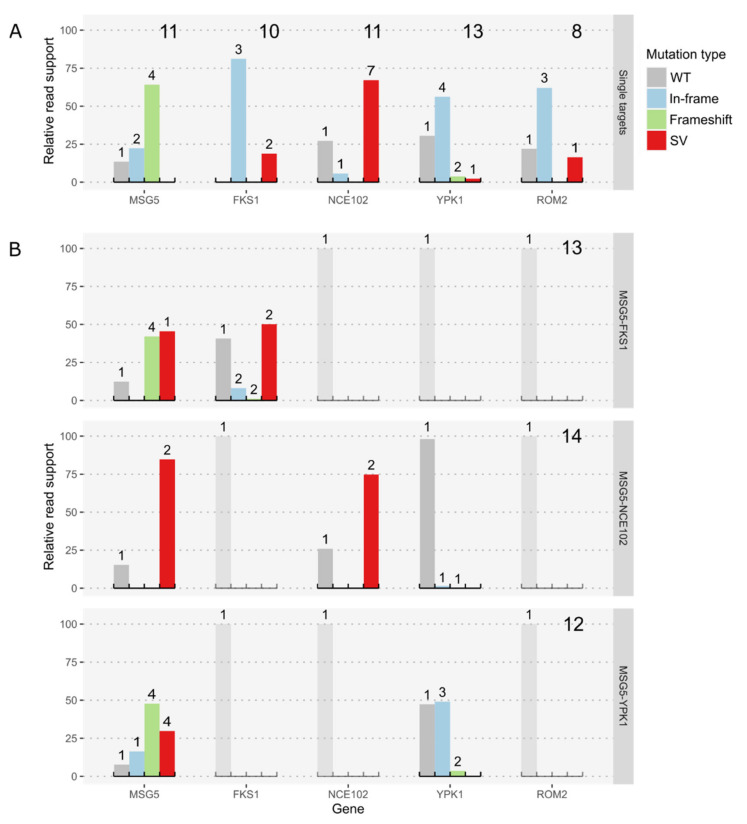
Comparison of on-target effects in single target (**A**) and double target experiments (**B**). Shown is the relative read support for the wildtype sequence and different groups of mutations. Large numbers on the right upper corners denote the number of sequenced colonies and the small numbers above bars denote the number of distinct genotypes per type of variant. Genotypes were classified into wildtype or no variant (WT), in-frame and frameshift InDels, including small Insertion and Deletions up to 50 bp, with a respective length of 3 nucleotides, or other length causing a shift of the reading frame (In-frame and Frameshift) and structural variants, including insertions and deletions of 50 bp and more or other kinds of structural variants (SV). (**A**) Summary of on-target mutations in single target CRISPR experiments. (**B**) Overview of on-target mutations in double target experiments with 2 targets each. The expected result for genes not targeted in an experiment is 100% of wildtype reads, therefore these subgraphs are dimmed.

The detailed analysis of the effects caused by targeting the single loci *MSG5*, *FKS1*, *ROM2*, *YPK1*, and *NCE102* using WGS revealed major target-specific differences in the type of mutations occurring. The inspection of double target experiments using the same gRNAs showed a shift towards more complex mutations. For the double target experiments on the platform strain, carrying a Hygromycin resistance within a region of putatively non-essential genes, we observed the deletion of around 100 kbp at the 3′ end of chromosome 3. For each experiment, we defined the relative read support for different groups of on-target genotypes. We differentiate between wildtype sequence or no mutation, small insertions or deletions of less than 50 bp, leading to either in-frame or frameshift deletions, and SVs, which include deletions and insertions of at least 50 bp and translocations, duplications, inversions, and chromosomal rearrangements (Figure 2). Targeting efficiency was defined as the summarized relative read support for all types of mutations.

#### 3.1.1. Single Target Transformations

**Figure 3:**
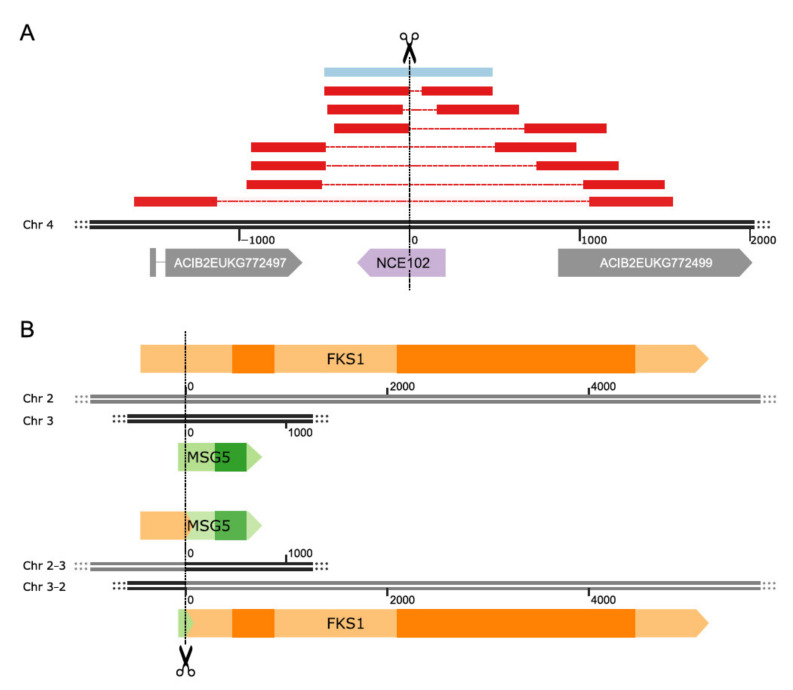
Detailed views of exemplary variants observed after CRISPR/Cas9-based strain engineering. (**A**) Overview of all deletion events at the *NCE102* locus, the red and blue bars indicate 1000 bp sequences upstream and downstream of the breakpoint aligned to the wildtype sequence. The blue bar corresponds to the detected in-frame deletion. The dashed lines represent the deleted regions. (**B**) The rearrangement of the *MSG5* and *FKS1* genes, with the wildtype genes at the top and the rearranged genes at the bottom. Darker regions within genes correspond to conserved domains.

The mutation efficiency at the different targets ranged from 70–100%. Perfect efficiency was observed for the *FKS1* locus, where none of the sequenced colonies showed the wildtype sequence at the targeted site (Figure 2A). Nonetheless, all identified mutations at this locus were in-frame mutations. Even the two SVs discovered (2 deletions: 267 and 441 bp, respectively) preserve the reading frame and the annotated conserved domains of the gene. Mutations in *YPK1* and *ROM2* are also almost exclusively in-frame, whereas in the *ROM2* target, we found no frameshift mutations (FS) at all, but a single deletion of 876 bp, which covers the translation start site and part of the promoter region. Within the *YPK1* target, 2 FS and 1 SV were detected, although one of the FSs was only supported by very few reads, and we were not able to confirm it by Sanger sequencing (Supplementary Results). The single SV is an 87 bp insertion, which duplicates Chr3:868,034-868,120; leading to an insertion of 29 amino acids (AAs). In contrast, mutations in the *MSG5* gene were mostly FS and only a few were in-frame mutations. *NCE102* also poses a special case since seven different SVs were identified and only a single colony showed a frameshift mutation. The SVs comprised seven deletions ranging from 69 to 2212 bp. The largest one caused partial deletions in the neighboring genes, ACIB2EUKG772497 and ACIB2EUKG772499. The first gene is a homologue of the *S. cerevisiae PHB1* gene, the second one is coding for a hypothetical protein (Figure 3A). All on-target mutations in the few colonies selected for BGS targets showed small frameshift deletions, except for the 3 bp deletion in the *BGS13* gene, which is in-frame.

#### 3.1.2. Double Target Transformations

Double target experiments revealed severe changes in the repair patterns compared to their single target counterparts. In particular, the repair of the DSB at the *MSG5* target changed drastically, showing a high number of SVs in all double target experiments (Figure 2B). All SVs detected in *MSG5-NCE102* strains were chromosomal rearrangements between the two targets, which are located on chromosomes 3 and 4, respectively.

The rearrangements led to stop codons in both potential new reading frames. Similarly, the most abundant SV in *MSG5-FKS1* strains was a chromosomal rearrangement between the targets *MSG5* and *FKS1,* which are located on chromosomes 3 and 2, respectively. In contrast to the SVs in *MSG5-NCE102* strains, this chromosomal rearrangement led to a valid reading frame coding for a potential fusion protein of a short 5′ region of *MSG5* and the 3′ end of *FKS1*, being expressed under the control of the *MSG5* promoter. This fusion would still contain the conserved regions of the *FKS1* gene. The counterpart of this fusion protein, which would be expressed under the control of the *FKS1* promoter, shows an early stop codon (Figure 3B). The existence of the described transcripts was confirmed by RNA-seq of one of the clones, which harbored the chromosomal rearrangement. The other SV observed in *FKS1* is a deletion of 57 bp, also conserving the reading frame of *FKS1*. Multiplexing of *MSG5* and *YPK1*, which are both located on chromosome 3, caused a considerable number of large deletions in *MSG5,* but did not lead to any SVs in *YPK1*. This was not too surprising since reading frame disruptions in *YPK1* were also rare in single target engineering experiments (Figure 2A). In the context of the gRNA efficiency, we can see a considerable decline in mutations at the *FKS1* locus and a minor decline at the *YPK1* locus. The targeting efficiencies at the *MSG5* locus remained above 80% for all experiments, and the ones at the *NCE102* locus were about 75% (Figure 2B).

#### 3.1.3. Double Target Transformations within a Non-Essential Region

**Figure 4:**
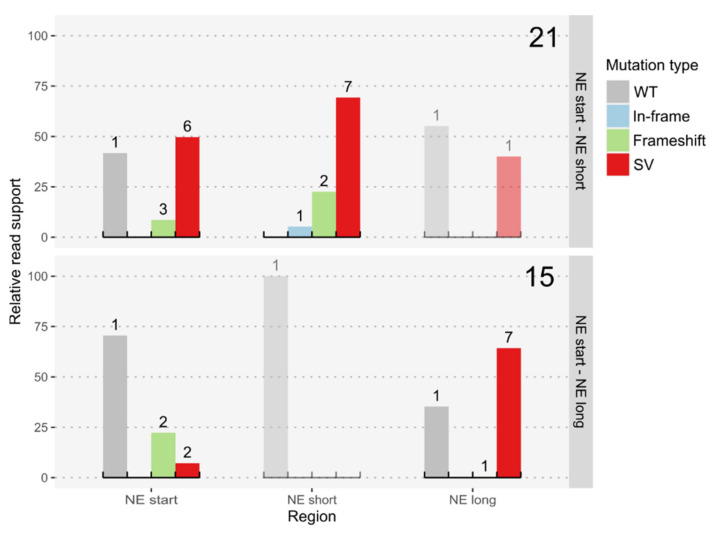
Relative read support for different mutation types at CRISPR/Cas9 targets in the strain *K. phaffii* BSYBG10_chr3ne_HygR. Large numbers on the right upper corners denote the number of sequenced colonies and the small numbers above bars denote the number of distinct genotypes per type of variant. Types of variants were classified into wildtype or no variant (WT), InDels, including small insertions and deletions up to 50 bp, with a respective length of 3 nucleotides, or other lengths causing a shift of the reading frame (In-frame & Frameshift), and structural variants, including insertions and deletions of at least 50 bp, and other kinds of SVs (SV). The SVs at the NE-long locus, in the strains where the NE start and NE short sites were targeted, indicate the complete loss of this region.

Introducing DSBs close to the Hygromycin resistance cassette (targets NE start–NE short, Figure 1B) led to about 40% of clones (32/83) being unable to grow on plates containing Hygromycin. The analysis of the WGS data showed that multiple clones lost the targeted region, but only two clones showed a clean knockout of the targeted region without additional or deleted bases (~2%). The remainder of the clones, not growing on Hygromycin containing plates, had lost the complete 3′ end of chromosome 3, starting either directly before or after the Hygromycin cassette (NE start, NE end). Both variants caused the loss of more than 100 kbp, containing 20 genes and an unknown number of rDNA repeats. The double target experiments targeting the beginning and end of the whole 80 kbp region of putatively NE genes (targets NE start–NE long, Figure 1B), in contrast, led to no loss of the Hygromycin resistance in any of the transformed colonies. However, many of the strains also lost the 3′ end of chromosome 3 with the encountered truncations starting up to 8 kbp upstream of the NE long target. One of the clones even showed an exact inversion of the complete NE region between NE start and NE long (Figure 4).

**Figure 5:**
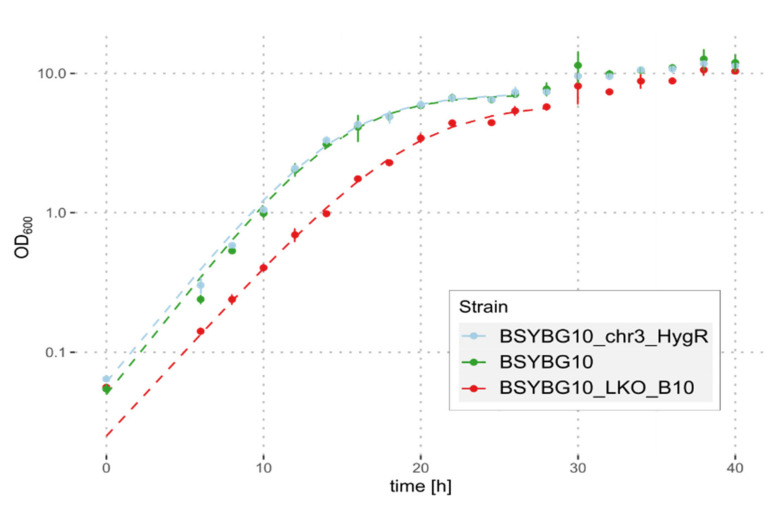
Growth of strains BSYBG10_LKO_B10, BSYBG10_chr3ne_HygR and the wildtype strain BSYBG10 in shake-flasks with glycerol as a carbon source. Mean values of 9 replicates are shown as dots and the error bars represent the standard deviation. Dashed lines represent the fitted growth curves based on the first 26 or 28 h, respectively.

We selected one of the clones, showing the 100 kbp truncation of chromosome 3, namely BSYBG10_LKO_B10, to further investigate and verify the genomic changes. A *de novo* assembly of Oxford Nanopore and Illumina sequencing reads confirmed the loss of the whole region of NE genes and revealed several telomeric repeats at the truncated 3′ end of chromosome 3, directly after the introduced DSB at the NE start target. Despite the large deletion in clone BSYBG10_LKO_B10, a comparable maximum cell density was reached by all three strains when grown in shake-flasks with glycerol as a carbon source. The maximum growth rate of BSYBG10_LKO_B10 was statistically significantly reduced as compared to the wildtype strain BSYBG10 (µ_max_ of 0.283 h^−1^ and 0.309 h^−1^, respectively; *p* = 0.05; one-tailed Wilcoxon signed rank test). The µ_max_ of the platform strain BSYBG10_chr3ne_HygR was 0.303 h^−1^ and did not significantly differ from the wildtype strain (Figure 5).

### 3.2. Off-Targeting Effects

We identified single nucleotide variants, InDels, and SVs compared to the reference genome for each sequencing run. To identify candidate *de novo* mutations (DNMs), we systematically filtered the called variants to exclude variants already present in the base strain and false positive calls caused by sequencing errors and errors in the reference sequence. We also excluded an SNV, which occurred in multiple transformants across experiments including different gRNAs, but which were all based on strain BSYBG10_3S1K_Calb. This resulted in 1 to 19 total DNMs per sequencing run (1–4 per single colony), which included already known on-target variants. Nonetheless, almost two-thirds (59/94) of off-target SNVs and InDels were within an annotated coding sequence, therefore potentially affecting protein function (Table 2). The identified SVs may still contain false positive calls caused by sequencing errors, but we also identified some high confidence off-target SVs. Of note is the insertion of 235 novel bases, which seem to stem from a species within the genus *Bacillus* (best BLASTN hit against nr/nt: *Bacillus luti*, NCBI: CP040336.1, 94% sequence identity). The analysis also suggested that two of the sequenced strains have a truncated 3′ end of chromosome 1, similar to the truncations caused by targeting the NE gene region at the 3′ end of chromosome 3.

**Figure 6:**
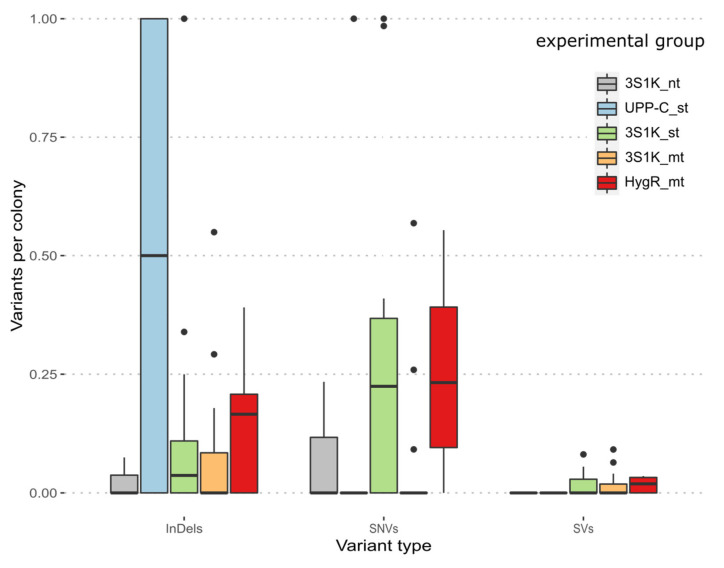
Boxplot of the relative frequency of variants per colony for pooled sequencing as well as single colony sequencing. Called variants were separated into the different experimental groups and included InDels, SNVs and SVs: the control group harboring no gRNA (3S1K_nt), single target groups (UPP-C_st, 3S1K_st) and the double target groups (3S1K_mt, HYGR_mt).

For a comparison of the frequency of off-target mutations occurring in the different types of CRISPR experiments, we grouped the sequencing results by platform strain and type of CRISPR/Cas9 plasmid used. This resulted in five groups, namely three experiments based on strain BSYBG10_3S1K_Calb, with CRISPR/Cas9 plasmids with no gRNA, one gRNA, and two gRNAs (3S1K_nt, 3S1K_st, 3S1K_mt), one experiment based on strain UPP-C using CRISPR/Cas9 plasmids with one gRNA (UPP-C_st) and one experimental group based on BSYBG10_chr3ne_HygR (HygR_mt). For all experimental groups, the relative frequency of off-target mutations per single colony is lower than one for InDels, SNVs, and SVs (Figure 6).

*In silico*, we identified potential off-target binding sites for the CRISPR/Cas9 complex for all used gRNAs. We allowed for a maximum of ten mismatches and no RNA or DNA bulges. Furthermore, potential off-target sites were identified using CHOPCHOP [82], which resulted in a single hit only, namely for the gRNA targeting the *YPK1* locus. Although 21 of 117 identified DNMs had a potential off-target binding site identified next to them, those off-target binding sites are unlikely to have caused CRISPR/Cas9 activity as they all showed at least 8 bases difference towards the used gRNA (Table 2). Moreover, for all but two of the identified unspecific binding sites, there were mismatches within the five bases next to the PAM motive (Supplementary Table S9), often referred to as the seed region [30], making an actual CRISPR/Cas9 related cleavage event at those sites even more unlikely. Furthermore, all the identified DNMs occurred only once, suggesting that these are random mutations happening during the transformation process, rather than unspecific CRISPR/Cas9 activity. To ensure that we did not miss any off-target CRISPR/Cas9 activity, we also determined off-target binding sites next to all unfiltered variants, with the result that even within unfiltered variants there were at least five bases difference of possible off-target binding sites towards the used gRNA. Additionally, we visually reviewed the off-target binding sites identified with CHOPCHOP and R with less than five mismatches in IGV but did not find any mutations in the mapped reads.

## 4. Discussion

We selected 146 CRISPR/Cas9 engineered single colonies transformed with a diverse set of CRISPR/Cas9 gRNAs for whole genome sequencing to investigate the effects of CRISPR/Cas9 induced DSBs followed by NHEJ. Our comparison of single target CRISPR/Cas9 transformations to double target experiments revealed a general high frequency of on-target SVs with a clear rise of on-target SVs as a response to multiplexing. The simultaneous introduction of two DSBs within a non-essential (NE) region even led to a truncation of the targeted chromosome 3, causing the loss of more than 100 kbp at the 5′ end of the chromosome.

### 4.1. On-Target Behavior

The determined efficiencies of 70–100% in single target CRISPR/Cas9 experiments correspond well with previous studies [6,48]. Nevertheless, *MSG5* was the only target predominantly showing frameshift mutations. This would be the intended behavior since a frame-shift mutation most likely renders the targeted gene non-functional. It would also be the expected behavior under the assumption that DSBs are preferably repaired by classical NHEJ (c-NHEJ). The repair events in *YPK1*, *FKS1*, and *ROM2* almost exclusively consisted of relatively small in-frame deletions. Since all of those mutations kept the reading frame of the genes intact and did not alter the conserved domains of the annotated genes, we assume that the resulting mutated proteins are still at least partially functional. Thus, the high occurrence of in-frame deletions and the absence of frame-shift deletions might be an indication that those genes are essential in *K. phaffii.* Furthermore, all chromosomal rearrangements in *MSG5-FKS1* double target experiments led to a *MSG5-FKS1* fusion gene, which is still transcribed and carries the functional conserved domain of *FKS1*, backing the theory that *FKS1* has an essential function. *FKS1* was recently marked as essential in a transposon integration study in *K. phaffii* strain GS115 [89] as well. In the same study, *YPK1* was interestingly tagged as “ambiguous”. *ROM2* was not analyzed, because it is not annotated in the reference sequence of *K. phaffii* GS115 [89,90]. The genes *YPK1*, *FKS1* and *ROM2* in *S. cerevisiae* are non-essential, but all three have a paralogue, making them redundant. Their double knockouts in *S. cerevisiae*, however, are inviable [55,56,57]. Alternatively, frameshift deletions in the aforementioned genes might have caused an unusual phenotype and therefore such colonies might not have been picked for analysis. For the BGS targets no mutation frequency was calculated, as only very few colonies were sequenced. The frameshifts in the targets *BGS5*, *BGS7*, and *BGS12*, as well as the in-frame deletion in *BGS13*, however, confirm the results of Cereghino et al. who reported insertions of integration cassettes in those open reading frames previously, while still reporting an intact reading frame for *BGS13* [51,52].

A reduction of targeting efficiencies for double target experiments, as previously reported [50], was only seen for *FKS1* and *YPK1*, both being putatively essential genes in *K. phaffii*. Thus, we theorize that multiplexing *per se* does not lead to a decrease in efficiency but leads to an increase of SVs. Those major genomic changes lead to less survivors, when targeting essential genes and, consequently, to a higher percentage of wildtype strains within these survivors. The rate of SVs caused by CRISPR/Cas9 transformations in other organisms was so far reported to be at most 20% [24]. This leaves the high rate of SVs we observed in *K. phaffii,* reaching up to 75% depending on the target and type of experiment, without precedence.

NHEJ, MMEJ, and HR are competing pathways for the repair of DNA double strand breaks [91]; c-NHEJ usually causes small InDels [92,93] and the relatively less studied MMEJ can lead to deletions up to several kb in size, as well as chromosomal rearrangements [94,95,96]. Because of the high number of large deletions and chromosomal rearrangements, especially in double target experiments, we speculate that the simultaneous introduction of two DSBs overwhelms the c-NHEJ pathway in *K*. *phaffii* and causes a rise in either MMEJ-induced repair events or an induction of an alternative NHEJ (a-NHEJ) pathway. Alternatively, the observed events might be simply a consequence of spatial separation of the generated DNA ends in case of multiplexing and further increasing the generally high tendency for SVs in *K. phaffii* that was also shown in studies on targeted integration [97]. The repair of DSBs via HR in wildtype *K. phaffii* strains requires large amounts of repair template with long homologous arms [6,38]. Since no such donor DNA was provided for the targeted sites, we do not believe that HR played an essential role in our experiments. In all chromosomal rearrangements, one of the two repair events shows a rather short and imperfect microhomology (5 bp with two mismatches). These microhomologies are clearly shorter than the MHs described as necessary for MMEJ in *S. cerevisiae* [98]. Nonetheless, MMEJ has been shown to be invoked by MHs as short as 2 bp [99] and since almost all chromosomal rearrangements showed the same repair pattern, we conjecture that those DSBs were most likely fused using MMEJ. Since repair events by a-NHEJ are relatively unpredictable and usually unwanted, the safer, but more laborious option would be to consecutively introduce frameshifts using CRISPR/Cas9, rather than simultaneously. Another solution to increase the number of frameshift mutations could be to overexpress the genes building the KU complex, as this has been shown to reduce MMEJ and increase c-NHEJ repair events in fission yeasts [100]. Alternatively, to avoid unwanted on-target effects, repair templates can be used to invoke HR in *K. phaffii*. This is especially successful if applied to an NHEJ deficient Δku70 strain [6]. Furthermore, the overexpression of HR genes in *K. phaffii* has recently shown convincing results. This strategy facilitated the simultaneous introduction of multiple genes, when using CRISPR in combination with a repair template for HR [38].

The simultaneous targeting of the flanking sequences of the NE region on chromosome 3 led to a very high number of chromosomal truncations, a puzzling phenomenon that has been previously seen in *K. phaffii* in a different context [38] and after CRISPR transformation of human cancer cell lines [101], but which has not been studied further. Chromosomal truncations by intent have been used to reduce the size of genomes of transgenic crops, but usually require the introduction of a template carrying the telomere repeats [102,103]. Our results, confirmed by long-read sequencing, suggest that *K. phaffii* is able to add telomeric repeats to a rogue DNA double strand break using an unknown type of rescue DNA repair mechanism. Notably, these truncation events seem to occur frequently and were even experienced at the end of chromosome 1, which was not targeted at all. This behavior could potentially be exploited to further minimize the *K. phaffii* genome based on strain BSYBG10_LKO_B10, since chromosome 1 and chromosome 4 also carry large regions with putatively NE genes at their 3′ ends. However, this would imply the loss of their ribosomal DNA repeats, which could impair protein translation and cell growth. Furthermore, *K. phaffii* would also make a good candidate for further study of this repair mechanism, because of the high frequency of truncation events.

The truncation of chromosome 3, together with the growth analysis, proved the identified genes to be non-essential under standard laboratory growth conditions. Nonetheless, the strain suffered from an initial growth deficiency, but reached a similar maximal growth after about three days including the preculture phase. This initial lag phase could be connected to the loss of the genes, as well as the partial loss of ribosomal DNA. *Komagataella phaffii* carries multiple rDNA repeats on the 3′ end of all four chromosomes, except chromosome 2. The total number of repeats of the rDNA cluster is estimated to be between 20 and 30 [72,104], but it is unknown how these are distributed between the chromosomes. Consequently, it is also unknown if the number of rDNA repeats per chromosome are stable and if the loss of the rDNA on one chromosome could be balanced by the increase of rDNA repeats on another chromosome, which would explain the initial lag in growth of the truncated strain BSYBG10_LKO_B10.

The visual examination of mapped reads at targeted loci revealed multiple mutations that were supported by a very low number of reads, indicating multiple genotypes per single colony. One explanation for this could be that the performed single colony streak outs were insufficient to perfectly separate clones. But since some of the WGS results showed up to five different genotypes for single colonies (Supplementary Table S5), we concluded that some of the clones simply had not yet lost the CRISPR plasmid when single colony streak outs were performed. Although this is not considered problematic, if CRISPR is used to study effects of a gene knockout based on a variety of clones, it can be of concern if single clones are used for further experiments. The simplest solution would probably be the introduction of a dilution step, followed by an additional single colony streak out on non-selective media to give the cells more time to lose the plasmids. In connection with these mixtures of colonies, we also encountered frameshift mutations within the *YPK1* locus, one of them with a read support of 50 reads (slightly lower than the ~80 reads expected per colony). Sanger sequencing of the locus led to inconclusive results for two of the colonies, even after an additional round of singulation. None of the colonies showed a clear frameshift mutation, indicating that those mutants can either not survive for long, or only within a mixture together with intact *YPK1* mutants.

### 4.2. Off-Targeting

The genome wide analysis of InDels, SNVs, and SVs showed low to no signs of CRISPR related off-targeting. No DNMs were identified at potential CRISPR off-target sites. Sequences of potential off-target binding sites of the CRISPR complex, identified close to DNMs, differed in at least eight bases to the used gRNA and most of them showed mismatches in the seed region of the gRNA. This largely complies with results from other studies [21,32]. Furthermore, no apparent rise in any type of variant could be identified in any of the experimental groups and—more importantly—no difference could be observed between the experiments with empty plasmids and the single target and double target experiments. Nonetheless, we observed a considerable number of mutations, which could potentially have an influence on protein function, but they all occurred only once, and they were randomly distributed across the genome. The sequencing of mixed colonies is prone to more false positive variant calls, due to sequencing errors and a lower read depth per colony, and, therefore, required more rigorous filtering. Nonetheless, we are confident that we did not filter out any relevant off-targeting events, as even in the unfiltered variant calls there are at least five mismatches between the identified putative binding sites and the used gRNA. Furthermore, the numbers of off-target DNMs in multi colony sequencing results are coherent with the ones in single colony sequencing.

This leads us to the conclusion that it is very unlikely to introduce systematic off-target variants into the genome of *K. phaffii* by CRISPR/Cas9 if the gRNAs are designed adequately. Therefore, if enough clones with confirmed frameshift mutations are considered for studying the effects of CRISPR/Cas9 mediated gene knockdowns, in our opinion, off-target effects are negligible. However, since the *K. phaffii* genome is very densely packed, with about 80% of the genome coding for proteins [62], unrelated random mutations often occur within gene coding regions. Furthermore, structural variations are relatively frequent especially at the target locus—even if just a single DNA site is targeted. Therefore, WGS is essential in case of functional studies of low numbers of clones or single strains where genes have been targeted by CRISPR mediated genome engineering approaches in *K. phaffii.*

## Figures and Tables

**Table 1:** Overview of genes targeted in the single target CRISPR/Cas experiments. “Name” denotes the gene names used in this study and are underlined if they relate to the homologous gene in *Saccharomyces cerevisiae*.

Locus Tag	Name	Potential Function/Role	References
ACIB2EUKG769938	*BGS5*	Heavy chain dynein	[52]
ACIB2EUKG768596	*BGS7*	Pleckstrin-like, nuclear transport	[52]
ACIB2EUKG769034	*BGS12*	Cytoplasmic dynein, intermediate chain	[52]
ACIB2EUKG770622	*BGS13 (PKC1)*	Pc1 kinase, a protein serine/threonine kinase, which controls a highly conserved signaling pathway managing cell wall integrity	[51,52,53,54]
ACIB2EUKG771351	*MSG5*	Dual-specificity protein phosphatase (i.e., Ser/Thr- and Tyr-specific), which plays a role in the regulation of at least two mitogen-activated protein kinase (MAPK)-mediated pathways	[55]
ACIB2EUKG771893	*YPK1*	Ser/Thr-protein kinase and is a relevant part of sphingolipid-mediated and cell integrity signaling pathways	[55,56]
ACIB2EUKG770030	*FKS1*	1,3-beta-D-glucan synthase, therefore being responsible for the synthesis of the polysaccharide, which is the main structural component of the cell wall	[57]
ACIB2EUKG771759	*ROM2*	One of three guanine nucleotide exchange factors probably specific to Rho1p and Rho2p	[55]
ACIB2EUKG772498	*NCE102*	Integral membrane protein, being involved in an alternative pathway for protein export	[58,59]

**Table 2:** Numbers of called variants, showing total calls, which include all called variants compared to the reference including variants already present in the base strain, versus candidate *de novo* mutations (DNMs), which only include filtered variants likely to have happened during or after transformation of the platform strain. DNMs were further separated into on-target and off-target mutations. SnpEff off-target calls are reduced to variants classified by SnpEff to be within an open reading frame. Potential CRISPR/Cas9 off-target sites include sites with up to 10 mismatches from the used gRNA. The colony type describes if the sequencing was performed on a single colony or on a mixture of 5 colonies. The base strains relate to BSYBG10_3S1K_CalB, BSYBG10_chr3ne_HygR, and UPP-C (3S1K_CalB, chr3n3_HygR and UPP-C) and “#colonies” denotes the total number of colonies included in each group.

Colony Type	CRIPR Plasmid	Base Strain (#Colonies)	Type	Total Calls	DNMs	DNMs On-Target	DNMs Off-Target	SnpEff Off-Target	CRISPR Off-Target *
**single**	**no gRNA**	3S1K_CalB (1)	SNPs	16	0	-	0	0	-
			InDels	65	0	-	0	0	-
			SVs	93	0	-	0	-	-
	**single gRNA**	UPP-C (6)	SNPs	91	1	0	1	1	0
			InDels	367	9	6	3	0	1
			SVs	224	0	0	0	-	0
		3S1K_CalB (3)	SNPs	52	2	0	2	2	0
			InDels	188	3	2	1	1	0
			SVs	190	1	1	0	-	0
	**two gRNAs**	3S1K_CalB (9)	SNPs	161	0	0	0	0	0
			InDels	661	4	4	0	0	0
			SVs	733	9	8	1	-	0
		Chr3ne_HygR (2)	SNPs	33	0	0	0	0	0
			InDels	131	1	1	0	0	0
			SVs	354	3	1	2	-	0
**mixed**	**no gRNA**	3S1K_CalB (10)	SNPs	268	1	-	1	0	-
			InDels	209	1	-	1	1	-
			SVs	284	0	-	0	-	-
	**single gRNA**	3S1K_CalB (50)	SNPs	1935	10	0	10	4	1
			InDels	1133	31	23	8	6	2
			SVs	1785	18	10	8		1
	**two gRNAs**	3S1K_CalB (30)	SNPs	898	10	0	10	4	3
			InDels	721	34	12	22	19	1
			SVs	933	21	15	6	-	2
		Chr3ne_HygR (35)	SNPs	2776	24	1	23	16	2
			InDels	698	28	16	12	5	3
			SVs	1870	24	18	6	-	5

* All potential binding sites identified next to DNMs differed by at least 8 bases from the used gRNA.

## Data Availability

All base-called sequence data are accessible under ENA project PRJEB54592 (Available online: http://www.ebi.ac.uk/ena/data/view/PRJEB54592 (accessed on 19 August 2022)).

## Supplementary Materials

The following supporting information can be downloaded at: https://www.mdpi.com/article/10.3390/jof8100992/s1, Supplementary Materials and Methods: Plasmid maps, Variant filtering and Sequencing of *YPK1* locus; Supplementary Tables: Supplementary Table S1: Overview of used and constructed strains and base-plasmids within this study; Supplementary Table S2: Overview of all ordered synthetic sequences for the construction of single target and double target CRISPR plasmids; Supplementary Table S3: Overview over all used primers; Supplementary Table S4: Detailed overview over all sequencing runs, including information about the target, the used gRNAs within the experiments, and the number of colonies in a sequenced library; Supplementary Table S5: Detailed counts of reads supporting each genotype in single target CRISPR/Cas9 experiments, based on strain BSYBG10_aox1_3S1K-CalB; Supplementary Table S6: Detailed counts of reads supporting each genotype in multi target CRISPR/Cas9 experiments, based on strain BSYBG10_aox1_3S1K-CalB; Supplementary Table S7: Detailed counts of reads supporting each genotype in multi target CRISPR/Cas9 experiments, based on strain BSYBG10_Chr3ne_HygR; Supplementary Table S8: List of all genes identified to be within the putatively non-essential region on the 5′ end of chromosome 3; Supplementary Table S9: Overview over all identified *de novo* mutations, with a possible CRISPR/Cas binding site in its proximity. Refs. [105,106,107,108,109,110,111] are cited in Supplementary Materials.
