# Supplementary material for: Whole Genome Sequencing Analysis of Effects of CRISPR/Cas9 in Komagataella phaffii: A Budding Yeast in Distress
Source: J Fungi (Basel). 2022 Sep 21;8(10):992. doi: 10.3390/jof8100992 (PMC9605565; doi:10.3390/jof8100992)
Supplement: Supplementary file 1 [file jof-08-00992-s001.zip › Supplementary_Methods_and_Results_JoF_final2.pdf]

Whole genome sequencing analysis of effects of CRISPR/Cas9 in *Komagataella phaffii*: A budding yeast in distress

Veronika Schusterbauer[1,2], Jasmin E. Fischer[1], Sarah Gangl[1], Lisa Schenzle[1], Claudia Rinnofner[1], Martina Geier[1], Christian Sailer[3], Anton Glieder[1], Gerhard G. Thallinger[3,4,*]

[1]*bisy GmbH., Wuenschendorf 292, 8200 Hofstaetten, Austria*
[2]*Institute of Medical Engineering, Graz University of Technology, Stremayrgasse 16, 8010 Graz, Austria*
[3]*Institute of Biomedical Informatics, Graz University of Technology, Stremayrgasse 16, 8010 Graz, Austria*
[4]*OMICS Center Graz, BioTechMed Graz, Stiftingtalstraße 24, 8010 Graz, Austria*

Supplementary Methods

Plasmid Maps

Supplementary Figures S1 – S3 show the plasmid maps of plasmids pBSY3S1K_intAOX1_CalB, pPpT4_pHTX-pHHF-PARS1-pILV5-hsCAS9 and plasmid pPpT4_pHTX-pHHF-PARS1-pTEF1-hsCAS9 respectively.

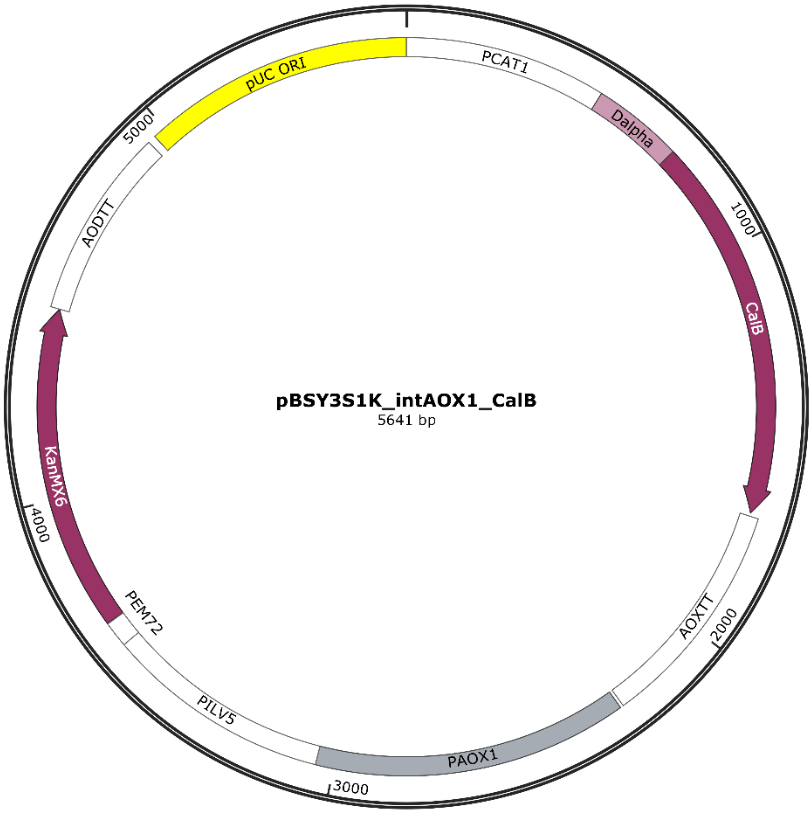

Supplementary Figure S1: Plasmid Map of plasmid pBSY3S1K_intAOX1_CalB for targeted integration of a CalB expression cassette under the control of the P_{CAT1} promoter into the AOX1 locus.

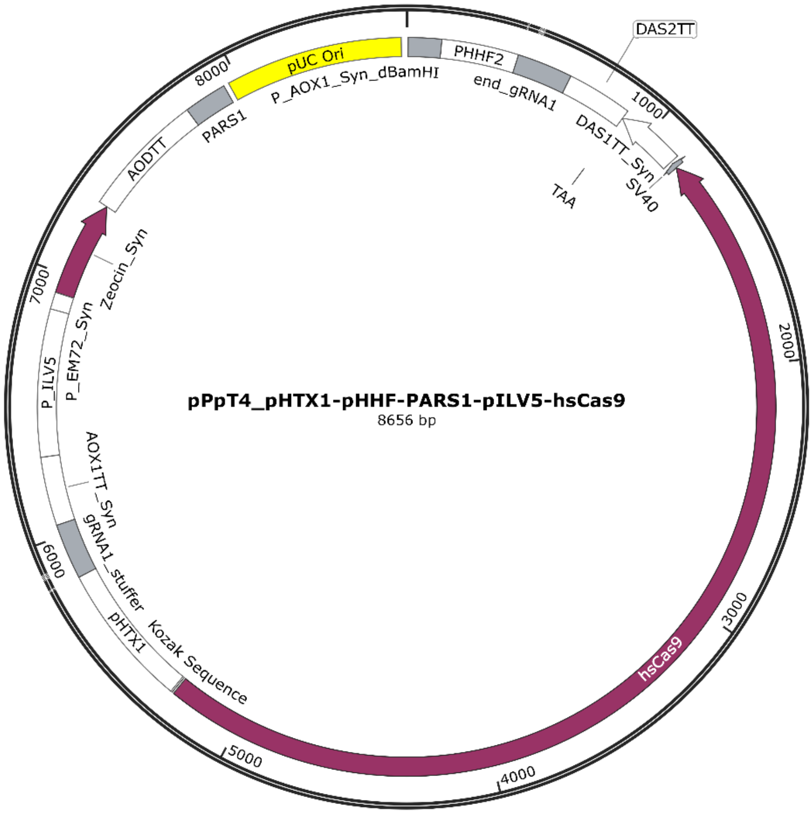

Supplementary Figure S2: Plasmid Map of the self-replicating plasmid pPpT4_pHTX-pHHF-PARS1-pILV5-hsCAS9 for multiplexed engineering of two targets. One gRNA expressed under P_{HTX1} promoter the other one under the P_{HHF} promoter. Zeocin resistance under the control of the control of the P_{ILV5} promoter.

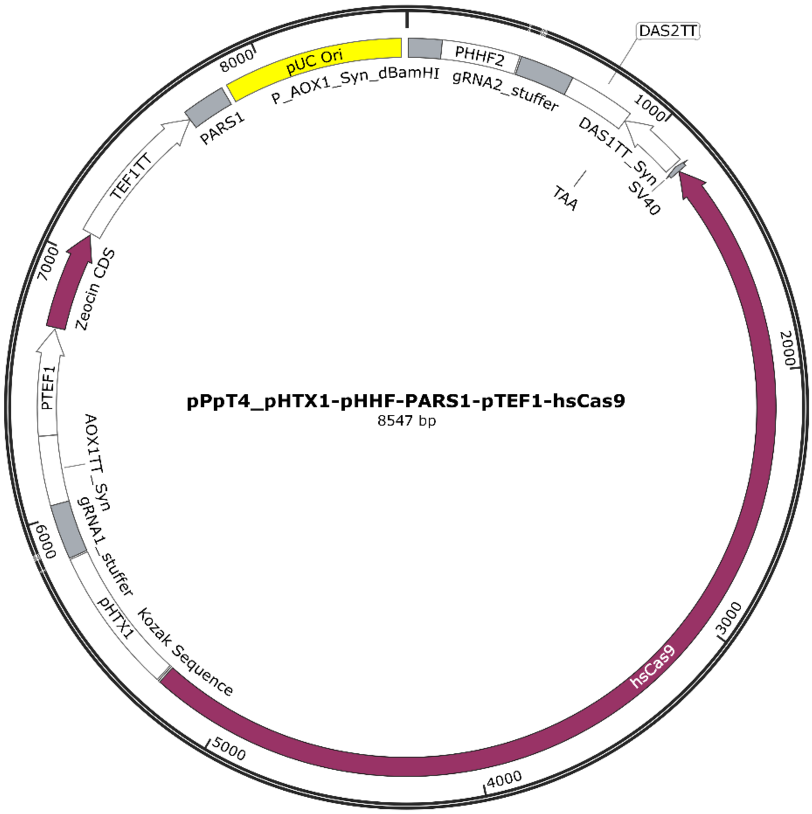

Supplementary Figure S3: Plasmid Map of the self-replicating plasmid pPpT4_pHTX-pHHF-PARS1-pTEF1-hsCAS9 for multiplexed engineering of two targets. One gRNA expressed under P_{HTX1} promoter the other one under the P_{HHF} promoter. Zeocin resistance under the control of the control of the P_{TEF1} promoter.

Variant filtering

All called variants, including single nucleotide variants (SNVs) and small insertions and deletions (InDels) as well as structural variants (SVs) were filtered in R. VCF files were read and modified, using the R package VariantAnnotation v1.38.0 (1) and StructuralVariantAnnotation v1.8.2 (2). First, variants already called in the base strain were filtered. In order to avoid counting variants twice, InDels showing more than 50 deleted or inserted bases were excluded from the InDel calls and SVs with less than or equal 50 inserted bases were also excluded. Furthermore, all variants were filtered based on low complexity and repeat regions. Telomeres, centromeres and rDNA regions were manually masked. Low complexity regions were called with dustmasker function from the NCBI blast+ (3) and extended for 10 bases up and downstream to account for inaccuracies in variant calling. Repeat regions were called with either RepeatMasker vopen-4.0.7 (4) using *Pichia pastoris* as species and RepBase version 20181026 or with the function DetectRepeats from the R package Decipher v2.20.0 (5, 6) (http://www2.decipher.codes/). SNV and InDels were filtered if they overlapped any of the defined regions, SVs were only filtered if the start or the end of the SV lay within one of the defined regions. The remaining variants were called complex variants. SNVs and InDels were filtered according to the GATK best practices (7) and based on multiple quality measures (Supplementary Table S10). SVs were filtered if they had the “LOW_QUALITY” (QUAL < 1000) or “NO_ASSEMBLY” (SV is not supported by any assembly) tag. All complex variants, including filtered and unfiltered ones, were visually inspected in IGV and cluster regions were defined to exclude all variants which appeared in clusters of filtered and unfiltered variants, since those are most likely caused by systematic errors in library prep or sequencing. Furthermore, one SNV was excluded that appeared in more than 10 different sequencing runs, which included colonies transformed with different gRNAs but always based on strain *K. phaffii* BSYBG10_3S1K-CalB.

Supplementary Table S10: Filter setting for SNVs and InDels. Variants, for which the stated condition is true, were filtered.

**Type**	**SNV**	**InDel**
**QUAL**	< 30	< 30
**SOR**	> 3	-
**FS**	> 60	> 60
**MQ**	< 40.0	-
**MQRankSum**	< -12.5	< -20.0
**ReadPosRankSum**	< -8.0	-

Summarizing variants

The estimated number of total variants per clone was calculated using the variant allele fraction (VAF), since sequencing multiple haploid clones resembles sequencing of a polyploid organism. The VAF for small variants and structural variants was calculated based on information provided in the GATK (8) and GRIDSS (9) VCF files, respectively. For InDels and SNVs, the VAF was calculated as the fraction of reads supporting the variant, divided by total number of reads mapped at the locus (Equation 1). For SVs, the VAF was calculated by dividing the sum of fragments supporting a breakend or a breakpoint, by the sum of all fragments (Equation 2).

Equation 1: Variant allele fraction for small variants, based on measures provided by the GATK VCF output. With AD_i … # reads supporting genotype i.

$$VAF_i = \frac{AD_i}{\sum AD}$$

Equation 2: Variant allele fraction for structural variants based on measures provided by GRIDSS. With VF … # fragments supporting variant, BVF … # fragments supporting a breakend, REF + REFPAIR … # of all fragments supporting the reference

$$VAF = \frac{VF + BVF}{VF + BVF + REF + REFPAIR}$$

Supplementary Results

Sequencing of YPK1 locus

The analysis of the on-target mutations in sequencing run YPK1_6-10, containing reads of a mixed culture of 5 single colonies, transformed with a CRISPR/Cas plasmid targeting the YPK1 locus, showed a considerable amount of reads which had a deletion of a single base. Since a frameshift mutation would most probably render the gene non-functional, we tried to confirm this mutation with Sanger sequencing of the YPK1 locus by Microsynth (Microsynth AG, Balgach, Switzerland). The sequencing of the 5 colonies in question revealed either a deletion of 3 bases or the wildtype sequence for 3 of the colonies. The sequence of the other 2 colonies, namely 2D and 2G stopped before the CRISPR target (Supplementary Figure S4A). Those two colonies were again split into multiple single colonies, by streaking them out on non-selective media. Subsequently 5 colonies were picked per plate and the YPK1 locus was Sanger sequenced from both sides by Microsynth. None of the 10 colonies showed the deletion of a single base, but still for two of the colonies the sequence stopped at the CRISPR target, indicating a mixture of different mutations at this locus (Supplementary Figure S4B & C).

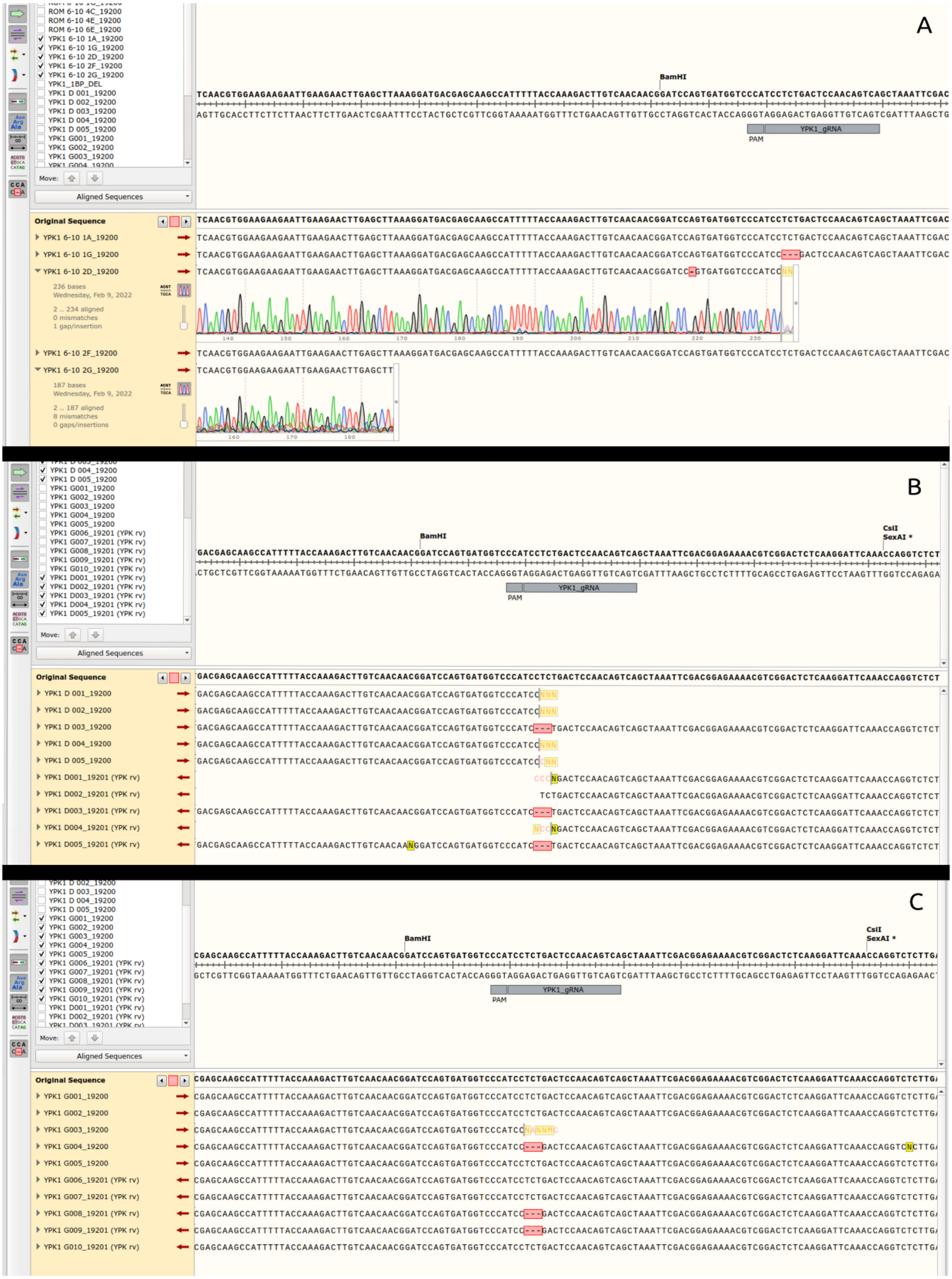

Supplementary Figure S4: SnapGene alignments of Sanger sequences of the YPK1 locus of colonies sequenced within sequencing run YPK1_6-10. A) Forward Sanger sequences of all 5 colonies within sequencing run YPK1_6-10, showing the premature stop of the Sanger sequences for colonies 2D and 2G. B) Forward and reverse sequences of 5 single colonies derives from colony 2D, showing a premature stop of the sequences in both directions exactly at the CRISPR target, for 3 of the 5 colonies. B) Forward and reverse sequences of 5 single colonies derives from colony 2G showing mostly wildtype sequences or deletions of 3 bases.

Supplementary References

1. Obenchain,V., Lawrence,M., Carey,V., Gogarten,S., Shannon,P., Morgan,M. (2014) VariantAnnotation: a Bioconductor package for exploration and annotation of genetic variants. *Bioinformatics*, **30**, 2076–8.
2. Cameron, D.L.; Dong, R.; Papenfuss, A.T. StructuralVariantAnnotation: a R/Bioconductor foundation for a caller-agnostic structural variant software ecosystem. *Bioinformatics* **2022**, *38*, 2046–2048.
3. Camacho,C., Madden,T., Coulouris,G., Avagyan,V., Ma,N. Agarwala,R. (2008) BLAST Command Line Applications User Manual. *https://www.ncbi.nlm.nih.gov/books/NBK279690*
4. Smith,A., Hubley,R., Green,P. (2013) RepeatMasker Open-4.0. *http://www.repeatmasker.org*
5. Wright,E.S. (2016) Using DECIPHER v2.0 to Analyze Big Biological Sequence Data in R. *R J.*, **8**, 352–359.
6. Schaper,E., Kajava,A. V., Hauser,A., Anisimova,M. (2012) Repeat or not repeat?—Statistical validation of tandem repeat prediction in genomic sequences. *Nucleic Acids Res.*, **40**, 10005.
7. Broad Institute (2018) Germline short variant discovery (SNPs + Indels). *https://software.broadinstitute.org/gatk/best-practices/workflow?id=11145*.
8. McKenna,A., Hanna,M., Banks,E., Sivachenko,A., Cibulskis,K., Kernytsky,A., Garimella,K., Altshuler,D., Gabriel,S., Daly,M., *et al.* (2010) The Genome Analysis Toolkit: A MapReduce framework for analyzing next-generation DNA sequencing data. *Genome Res.*, **20**, 1297–1303.
9. Cameron,D.L., Schröder,J., Penington,J.S., Do,H., Molania,R., Dobrovic,A., Speed,T.P., Papenfuss,A.T. (2017) GRIDSS: sensitive and specific genomic rearrangement detection using positional de Bruijn graph assembly. *Genome Res.*, **27**, 2050–2060.
